# Supplementary material for: Patient Preferences or Provider Pressure? The Relationship Between Coercive Contraceptive Care and Preferred Contraceptive Use
Source: Healthcare (Basel). 2025 Jan 14;13(2):145. doi: 10.3390/healthcare13020145 (PMC11765023; doi:10.3390/healthcare13020145)
Supplement: Supplementary file 1 [file healthcare-13-00145-s001.zip › Supplementary Table S1.pdf]

Supplementary Table S1. Logistic regression of preferred contraceptive method(s) use regressed on any coercion at last contraceptive counseling among a 2023 U.S. sample of reproductive-aged people assigned female at birth who had ever talked to a healthcare provider about birth control (*N*=1,197).

	Model 1	Model 2
	**Odds Ratio (95% Confidence Interval)**	**Odds Ratio (95% Confidence Interval)**
Any coercion at last contraceptive counseling	0.59 (0.40, 0.86)**	0.63 (0.42, 0.94)*
Sociodemographic controls		
Age	-	1.00 (0.98, 1.03)
Education level (ref: high school or less)		
Associate degree or some college	-	0.75 (0.43, 1.31)
Bachelor's degree	-	0.88 (0.50, 1.54)
Graduate school	-	1.44 (0.72, 2.86)
Insured (ref: uninsured)	-	1.09 (0.63, 1.89)
Race (ref: Non-Hispanic white)		
Hispanic	-	0.45 (0.27, 0.76)**
Non-Hispanic Asian	-	0.80 (0.42, 1.54)
Non-Hispanic Black	-	1.13 (0.68, 1.86)
Non-Hispanic mixed race	-	0.99 (0.42, 2.32)
Sexual orientation (ref: heterosexual)		
Bisexual	-	0.74 (0.49, 1.11)
Gay or lesbian	-	2.99 (0.90, 9.96)
Asexual, pansexual, queer, questioning, or prefer to self-describe	-	0.65 (0.39, 1.09)
Married (ref: unmarried)	-	1.44 (0.96, 2.17)
Time since last contraceptive counseling (ref: in the past year)		
1–3 years	-	0.68 (0.46, 0.99)*
4–6 years	-	0.48 (0.29, 0.80)**
7 years or more	-	0.85 (0.41, 1.76)

Notes: $^*p<0.05$, $^{**}p<0.01$, $^{***}p<0.001$.
