# Supplementary material for: Patient Preferences or Provider Pressure? The Relationship Between Coercive Contraceptive Care and Preferred Contraceptive Use
Source: Healthcare (Basel). 2025 Jan 14;13(2):145. doi: 10.3390/healthcare13020145 (PMC11765023; doi:10.3390/healthcare13020145)
Supplement: Supplementary file 1 [file healthcare-13-00145-s001.zip › Supplementary Table S2.pdf]

Supplementary Table S2. Logistic regression of preferred contraceptive method(s) use regressed on downward coercion at last contraceptive counseling among a 2023 U.S. sample of reproductive-aged people assigned female at birth who had ever talked to a healthcare provider about birth control (*N*=1,197).

	Model 1	Model 2
	**Odds Ratio (95% Confidence Interval)**	**Odds Ratio (95% Confidence Interval)**
Downward coercion at last contraceptive counseling	0.37 (0.20, 0.70)**	0.39 (0.20, 0.75)**
Sociodemographic controls		
Age	-	1.01 (0.98, 1.03)
Education (ref: high school or less)		
Associate degree or some college	-	0.73 (0.42, 1.28)
Bachelor's degree	-	0.87 (0.49, 1.52)
Graduate school	-	1.42 (0.71, 2.83)
Insured (ref: uninsured)	-	1.09 (0.63, 1.90)
Race (ref: Non-Hispanic white)		
Hispanic	-	0.44 (0.27, 0.74)**
Non-Hispanic Asian	-	0.80 (0.42, 1.55)
Non-Hispanic Black	-	1.13 (0.69, 1.87)
Non-Hispanic mixed race	-	0.96 (0.41, 2.26)
Sexual orientation (ref: heterosexual)		
Bisexual	-	0.74 (0.49, 1.12)
Gay or lesbian	-	2.88 (0.87, 9.57)
Asexual, pansexual, queer, questioning, or prefer to self-describe	-	0.66 (0.39, 1.11)
Married (ref: unmarried)	-	1.43 (0.95, 2.15)
Time since last contraceptive counseling (ref: in the past year)		
1–3 years	-	0.66 (0.45, 0.97)*
4–6 years	-	0.48 (0.29, 0.81)**
7 years or more	-	0.83 (0.40, 1.72)

Notes: *$p<0.05$, **$p<0.01$, ***$p<0.001$.
