# Supplementary material for: Patient Preferences or Provider Pressure? The Relationship Between Coercive Contraceptive Care and Preferred Contraceptive Use
Source: Healthcare (Basel). 2025 Jan 14;13(2):145. doi: 10.3390/healthcare13020145 (PMC11765023; doi:10.3390/healthcare13020145)
Supplement: Supplementary file 1 [file healthcare-13-00145-s001.zip › Supplementary Table S3.pdf]

Supplementary Table S3. Logistic regression of preferred contraceptive method(s) use regressed on upward coercion at last contraceptive counseling among a 2023 U.S. sample of reproductive-aged people assigned female at birth who had ever talked to a healthcare provider about birth control (*N*=1,197).

	**Model 1**	**Model 2**
	**Odds Ratio (95% Confidence Interval)**	**Odds Ratio (95% Confidence Interval)**
Upward coercion at last contraceptive counseling	0.67 (0.44, 1.02)	0.73 (0.47, 1.13)
Sociodemographic controls		
Age	-	1.01 (0.98, 1.03)
Education (ref: high school or less)		
Associate degree or some college	-	0.77 (0.44, 1.34)
Bachelor's degree	-	0.88 (0.51, 1.55)
Graduate school	-	1.45 (0.73, 2.88)
Insured (ref: uninsured)	-	1.11 (0.64, 1.92)
Race (ref: Non-Hispanic white)		
Hispanic	-	0.46 (0.27, 0.76)**
Non-Hispanic Asian	-	0.79 (0.41, 1.53)
Non-Hispanic Black	-	1.09 (0.67, 1.80)
Non-Hispanic mixed race	-	0.99 (0.42, 2.33)
Sexual orientation (ref: heterosexual)		
Bisexual	-	0.73 (0.49, 1.10)
Gay or lesbian	-	2.93 (0.88, 9.74)
Asexual, pansexual, queer, questioning, or prefer to self-describe	-	0.65 (0.39, 1.09)
Married (ref: unmarried)	-	1.45 (0.96, 2.18)
Time since last contraceptive counseling (ref: in the past year)		
1–3 years	-	0.67 (0.45, 0.98)*
4–6 years	-	0.47 (0.28, 0.78)**
7 years or more	-	0.83 (0.40, 1.70)

Notes: *$p<0.05$, **$p<0.01$, ***$p<0.001$.
